# Tree-Code Based Improvement of Computational Performance of the X-ray-Matter-Interaction Simulation Tool XMDYN

**DOI:** 10.3390/molecules27134206

**Published:** 2022-06-30

**Authors:** Michal Stransky, Zoltan Jurek, Robin Santra, Adrian P. Mancuso, Beata Ziaja

**Affiliations:** 1 European XFEL, Holzkoppel 4, 22869 Schenefeld, Germany; adrian.mancuso@xfel.eu; 2 Institute of Nuclear Physics, Polish Academy of Sciences, Radzikowskiego 152, 31-342 Kraków, Poland; beata.ziaja-motyka@cfel.de; 3 Center for Free-Electron Laser Science CFEL, Deutsches Elektronen-Synchrotron DESY, Notkestr. 85, 22607 Hamburg, Germany; robin.santra@cfel.de; 4 The Hamburg Center for Ultrafast Imaging, Luruper Chaussee 149, 22761 Hamburg, Germany; 5 Department of Physics, Universität Hamburg, Notkestr. 9-11, 22607 Hamburg, Germany; 6 Department of Chemistry and Physics, La Trobe Institute for Molecular Science, La Trobe University, Melbourne 3086, Australia

**Keywords:** X-rays, FELs, single particle imaging, molecular dynamics, tree algorithms

## Abstract

In this work, we report on incorporating for the first time tree-algorithm based solvers into the molecular dynamics code, XMDYN. XMDYN was developed to describe the interaction of ultrafast X-ray pulses with atomic assemblies. It is also a part of the simulation platform, SIMEX, developed for computational single-particle imaging studies at the SPB/SFX instrument of the European XFEL facility. In order to improve the XMDYN performance, we incorporated the existing tree-algorithm based Coulomb solver, PEPC, into the code, and developed a dedicated tree-algorithm based secondary ionization solver, now also included in the XMDYN code. These extensions enable computationally efficient simulations of X-ray irradiated large atomic assemblies, e.g., large protein systems or viruses that are of strong interest for ultrafast X-ray science. The XMDYN-based preparatory simulations can now guide future single-particle-imaging experiments at the free-electron-laser facility, EuXFEL.

## 1. Introduction

X-ray free-electron lasers (XFELs) provide X-ray pulses both of ultrahigh peak brightness and, simultaneously, of ultra-short pulse duration ranging from a few up to a few tens of femtoseconds. Single-particle imaging (SPI) experiments, performed at various XFEL facilities, aim to exploit these unique pulses to determine the structure of single, non-crystalline biological molecules [1], constantly progressing to improve the imaging resolution [2,3,4]. However, a key goal, i.e., the resolution at length scales of a few Angstroms ($10^{-10}$ m), has not yet been realized.

One fundamental obstacle is the XFEL-pulse induced radiation damage, triggered by photoionization and subsequent secondary processes on femtosecond timescales. Radiation damage alters a sample’s scattering strength on the few femtosecond timescale and triggers its subsequent disintegration on the few tens of femtoseconds timescale. Following the analysis performed in [1], only very intense and ultrashort X-ray pulses can image the sample before radiation-induced damage will significantly alter and ultimately destroy it. However, radiation damage is not the only limiting factor for SPI. Weak scattering from only a single molecule also contributes to the challenge of interpreting SPI data. Therefore, during an SPI experiment, a large number of two-dimensional diffraction patterns (many thousands, or perhaps even millions) from ‘identical’ particles (e.g., molecules, clusters or viruses) need to be recorded, in order to provide sufficient statistics, necessary to reconstruct a ‘meaningful’ average particle. Since the orientation of the sample with respect to the beam and the detector is unknown, the individual patterns must be oriented and merged into a three-dimensional diffraction volume (using dedicated algorithms) [5,6] before the three-dimensional electron-density map is reconstructed via phase retrieval [7]. These limitations of X-ray FEL imaging to resolving the structure of single macromolecules in a SPI experiment have been discussed in detail by, e.g., Fortmann-Grote et al. [8].

As numerous diffraction patterns are needed for a successful 3D reconstruction of a macromolecule [9], here the advantage of the FEL facilities based on superconducting technologies—and hence offering high repetition rate X-ray pulses—can be seen. Among such facilities are the European XFEL [10], LCLS II [11], and the Shanghai High Repetition Rate XFEL [12]. The superconducting accelerator technology enables generating from tens of thousands up to a million light flashes per second, which, in turn, makes it possible to record the required high number of diffraction images within a feasible experiment duration.

As emphasized above, while several single-particle-imaging (SPI) experiments with FELs have already been performed, e.g., [9,13,14], the ultimate goal of atomic resolution in such experiments has not yet been reached. Computational studies can guide future SPI experiments towards atomic resolution, e.g., by optimizing experimental parameters, establishing conditions for a successful sample reconstruction, by background evaluation, etc. That is why such computational studies are highly valuable for SPI experiments.

In order to explore the potential of the European XFEL for single-particle imaging, a comprehensive simulation platform, SIMEX [15], for SPI experiments has been developed [8,16]. This framework enables a realistic simulation of a single-particle imaging experiment at an XFEL facility, including source parameters, propagation of the coherent X-rays through optical elements, interaction of the photons with the imaged sample, detection of scattered photons, and structure determination. The tool has a modular structure consisting of: (i) multidimensional simulation of the X-ray source; (ii) simulation of the wave-optics propagation of the coherent XFEL beams; (iii) atomistic modelling of photon–matter interaction; (iv) simulation of the time-dependent diffraction process, including incoherent scattering; (v) assembling noisy and incomplete diffraction intensities into a three-dimensional data set; and (vi) phase retrieval to obtain structural information [16]. The SIMEX platform has been used in [8] to estimate the optimal pulse duration for an X-ray pulse of 5 keV photon energy (feasible at the SPB/SFX instrument of the European XFEL) to image reproducible, biological molecules. The platform has also been recently used in [17] to study imaging of hydrated proteins.

The bottleneck of the SIMEX simulations is the size of the studied system. The code used for atomistic modelling of photon–matter interaction within the SIMEX platform is XMDYN. It is a molecular-dynamics- and Monte-Carlo-based code for modelling X-ray driven dynamics in complex systems [18,19,20]. XMDYN was originally used only for small systems, containing up to several thousand atoms. It has been run by computing all mutual binary interactions among sample constituents. However, such an approach becomes computationally inefficient with increasing number of particles in the sample, ultimately disabling imaging studies of ‘realistic’ samples, using the SIMEX platform. Realistic samples mean here samples large enough to yield a sufficiently high scattering signal during their imaging.

This manuscript reports on technical improvements in the XMDYN code necessary to simulate X-ray radiation damage in larger systems such as large proteins, ribosomes, and viruses which are of strong interest for single particle imaging. In the manuscript, two new extensions of the XMDYN are discussed. The first one is the application of the Barnes–Hut-method based algorithm to create a more efficient computational module calculating secondary ionization in large molecular systems. This result is an original result. With this approach, we can now simulate secondary ionization in systems containing hundreds of thousands of atoms and ions. The second result, never reported before, is the substitution of the brute-force Coulomb solver in XMDYN with the tree-code based Coulomb solver PEPC (developed by Jülich Forschungszentrum) [21] which significantly improved the efficiency of Coulomb force calculations in XMDYN. In the paper, it is also shown in detail how the improvements performed have increased the code efficiency. This yields a very positive prospect for computational imaging studies of larger samples such as large protein systems or single viruses. Such simulations can guide future single-particle-imaging experiments at the EuXFEL.

## 2. Materials and Methods

### 2.1. XMDYN Code

For the simulation, the XMDYN, a molecular-dynamics- and Monte-Carlo-based code for modelling X-ray-matter interaction [18,19,20] was used. It is coupled on-the-fly to the atomic structure calculation tool, XATOM, [18,22,23]. This many-body and fully non-equilibrium model takes into account all relevant X-ray induced processes in matter (such as atomic photoionization, inner-shell Auger and fluorescent decay, collisional ionization and recombination), using their microscopic description. Chemical bonds can be represented using classical force fields [19,24]. However, in the current study, they were not included. XMDYN simulations follow the temporal evolution of a stochastically ionized system. The code has been successfully applied to describe the interaction of clusters and macromolecules with X-rays [18,20,24,25].

In the code, all atoms and free electrons are represented as classical particles in real space. The electronic structure is approximated by those of the corresponding isolated atoms or ions. The code follows the changes of the occupation number of each atomic orbital as a result of ionization or recombination events.

In order to follow time evolution of irradiated atomic assemblies, the XMDYN code uses a finite timestep propagation. A Monte Carlo algorithm is used to decide whether an ionization event occurs within a time step or not. After an ionization event, a new classical particle, i.e., an ionized electron is created, with an appropriate kinetic energy. The charge of the parent atom or ion is then increased by 1, while at the same time the occupation number of the atomic orbital included in the process is decreased by 1. Recombination is, naturally, the inverse process, i.e., the classical electron particle involved is removed, while the ion charge and the orbital occupation number involved are changed by −1 and +1, respectively. Charged particles interact with each other through Coulomb forces. The real-space propagation of the classical particles is performed with Molecular Dynamics.

For the time propagation, the main loop of the XMDYN code is responsible. Within one cycle of the loop, different modules (blocks) are executed consecutively to account for different physical processes. These blocks are the following:

1. *Recombination (RE) Block.* Within this block, all classical electron–ion pairs are analyzed in a search for configurations when an electron stays in the vicinity of an ion and satisfies the conditions for a recombination event to occur.
2. *Secondary Ionization (SI) Block.* Within this block, all classical electron–ion pairs are analyzed in a search for configurations when an electron stays in the vicinity of an atom or ion and satisfies the conditions for a secondary ionization event to occur.
3. *Monte Carlo (MC) Block.* Tracking of atomic processes, i.e., photoionization, Auger decay and fluorescence, depends on the probabilities of such events during a single time step. These probabilities are derived from atomic cross section and rates calculated by the ab initio XATOM code [18,22]. For each atom or ion, a random number is generated, which determines which event occurs during a single time step.
4. *Molecular Dynamics (MD) Block.* Within this block, all classical particles, i.e., the atoms, ions and classical electron particles are propagated in real space, during a single time step. XMDYN uses for the propagation the well-known velocity Verlet algorithm [26].

A more detailed discussion of the code structure can be found in Ref. [18]. XMDYN, together with XATOM, is a part of the software package XRAYPAC [27].

### 2.2. Computational Bottlenecks in XMDYN

For particle-based simulations, computational efficiency of different blocks in a respective code can be typically classified according to its dependence on the number of particles. Within the XMDYN code, the calculations in the MC Block scale linearly with the number of atoms and ions in the sample. In contrast, the implementations of the MD, SI and RE Blocks contain procedures that scale nonlinearly with the number of involved particles. Overall, these procedures are responsible for most of the computational time spent during a simulation of a large system.

The procedures in each block that mostly affect the computational efficiency are listed below:

1. MD Block: Evaluation of long-range Coulomb interactions between all charged particle pairs, both for force and potential calculations. In case of the most straightforward implementation (i.e., with two nested loops, both running over the number of charged particles in the sample, $N_{q}$), the computational time scales as, $t∼N_{q}^{2}$. This computational strategy is also called the ‘brute-force’ method.
2. SI block: In XMDYN, the occurrence rate of a secondary ionization event for a free electron and an atom/ion depends on the relative distance and velocities of these particles [18]. The decision regarding whether SI takes place or not requires their detailed analysis. In general, all electron–atom/ion pairs have to be analyzed within a time step. Therefore, in case of a straightforward (i.e., ‘brute-force’) implementation of secondary ionization, the computational cost, *t*, scales with the product of the number of free electrons and the number of atoms and ions: $t∼N_{e}\times N_{a}$.
3. RE block: A decision whether an electron recombines with an ion is also based on their relative distance and the velocities of the electron–ion pair [18]. Therefore, in the brute-force implementation the computational cost scales with the product of the number of electrons and number of ions: $t∼N_{e}\times N_{i}$, similarly as within the SI Block.

The computational costs can be significantly reduced by using the so-called tree-algorithm methods [21,28,29], which change the high-order power-law scaling with the number of particles to a more favourable one. The implementation of such methods in the MD and SI/RE Blocks will be discussed below.

### 2.3. Incorporating the PEPC Tree-Based Coulomb Solver into XMDYN

The Coulomb interaction between charged particles is a long-range interaction. Generally, it can have an effect on all particles within the simulation volume. Therefore, one should consider all pairwise combinations of charged particles to evaluate acting forces or potentials. Such an approach has an unfortunate drawback: one needs to perform $O(N_{q}^{2})$ computational steps to evaluate the forces acting on all $N_{q}$ particles. Hence, for large $N_{q}$ values, the calculations become computationally too expensive. However, there exist more sophisticated approaches that can tackle the computational complexity, still yielding values for forces and mean potentials that are accurate enough. One of them is the Barnes–Hut tree algorithm [28], which calculates the net effect of a group of charges on a target charge, using multipole expansion for their Coulomb interaction. Grouping of charges within an arbitrary system requires a dedicated analysis of particle locations. The Barnes–Hut method offers for this an efficient tree algorithm. Instead of developing our own solver, we chose to incorporate an available high-performance, tree-based Coulomb solver, PEPC [21,30] into the MD Block of XMDYN. The reduction of the computational complexity with the tree code also reduces to some extent the precision of Coulomb force or potential calculations. However, the accuracy can be controlled by the so-called angle parameter [21].

### 2.4. Tree Algorithm Developed to Speed-Up Secondary Ionization Calculation

As explained above, the decision made in the SI Block of the XMDYN code, whether a secondary ionization event can happen at a given timestep for an electron–atom/ion pair, requires a computationally expensive analysis of the mutual particle distances and velocities. In order to reduce the computational effort, a pre-assessment scheme for electron–atom/ion pairs based on their relative distance has been applied, i.e., any further evaluation of the secondary ionization criteria is carried out only if an atom or ion stays within a specified cutoff distance from a selected electron. From a computational perspective, the straightforward implementation of secondary ionization in molecular dynamics suffers similar efficiency problems as the calculation of Coulomb forces. The algorithm has to find the atoms/ions which are in a close proximity to a given electron. This procedure has to be repeated for all electrons. Overall, at each timestep, one has to perform $O(N_{e}\times N_{a})$ calculations, where $N_{e}$ is the total number of electrons, and $N_{a}$ is the total number of atoms and ions. Instead, if one organizes the atoms and ions into an ‘oct-tree’ [21], one can speed up this calculation to $O(N_{e}\log\left(N_{a}\right))$. We wrote a dedicated tree code for this purpose. An array implementation is used to represent the tree, similarly as in [29], however, the bit-length of each coordinate index is based on the maximum depth of the tree. It is then not a fixed number as in [29]. For example, if the chosen maximum depth of the tree is 5, then 5 bits are used for each coordinate (*x*, *y*, *z*). It yields a 3×5=15 bit index to identify the smallest box in which a particle might be located.

At each time step a Barnes–Hut tree is created for atoms and ions. A simulation box is defined so as to contain all these particles. Principally, the simulation box is then subdivided into eight orthorhombic sub-boxes (geometrically similar to the parent box). This occurs recursively, multiple times up to a maximum depth level, creating an oct-tree. Each atom or ion receives an identification number (following the idea in Ref. [29]) indicating its position in the tree (for more details, see Appendix A). The particle array is then sorted, using a quicksort algorithm, according to the identification numbers. A supporting structure is built, in order to allow for an efficient searching within the tree. This algorithm is based on Ref. [29] with some modifications mentioned in the previous paragraph and with different supporting structures which facilitate searching. The overall computational complexity of the tree building is $O(N_{a}\cdot log\left(N_{a}\right))$. However, the benchmarking of the tree-building step showed that it has a negligible time cost, when compared to the subsequent tree searching or to other computations performed outside the secondary ionization module. The most computationally expensive procedure of the secondary ionization module is actually the search of the closest atom or ion, performed for each electron. For each electron, it requires $O(log\left(N_{a}\right))$ computations. Therefore, for all electrons, it is $∼O(N_{e}\cdot log\left(N_{a}\right))$. With the tree building added, one gets the overall computational complexity of the algorithm equal to $O(\left(c_{1}\cdot N_{a}+c_{2}\cdot N_{e}\right)\cdot log\left(N_{a}\right))$, where $c_{1}$ and $c_{2}$ are some numerical coeffcients. However, as mentioned before, the coefficient $c_{1}$ turns out to be very small. The search for nearest neighbors of each electron is limited to a maximum distance estimated from the interaction cross-section. The tree is searched recursively, at each level eliminating sub-boxes that are “outside” of the maximum distance. For more details, see the Appendix A.

## 3. Results

### 3.1. Improved Coulomb Force Calculations

The improved XMDYN has been tested on a simple study system: finite water cubes of different sizes (see Table 1) irradiated with a 7.12 keV free electron laser pulse of 15 fs FWHM duration at a fluence of $3.5\times 10^{12}$ photons/μm${}^{2}$. A one-attosecond long timestep was used for the simulations. The starting time point was set at the pulse center. The initial system configuration was precalculated for each sample size, in order to have the same proportion between the total number of atoms and ions and the total number of free electrons for each sample size, which is necessary to meaningfully evaluate the impact of the tree algorithm on the efficiency of Coulomb interaction calculations.

Although the efficiency of the applied Coulomb solver, PEPC, was already tested (as reported earlier in, e.g., [21]), for the sake of completeness, the efficiency records for our study case are also listed below. Table 2 lists the computational cost in seconds when performing a single attosecond simulation timestep. The original ‘brute-force’ solver performs quite well for small samples. The single threaded tree code overtakes the brute-force solver only at a sample size of 75 Å. The tree code run with four parallel tree-walkers is already faster than the brute-force solver for the sample size of 47 Å. The performance depends on the chosen value of the angle parameter, θ, in the tree code [21]. The PEPC manual recommends θ values between 0.1 and 0.6. Higher values imply faster computation but less accuracy. Here, the value of θ=0.4 was used which gives accurate results for force and potential calculations at reasonable computation speed.

### 3.2. Improvement of Secondary Ionization Calculation

For the calculations shown in the previous section (Table 2), XMDYN’s default secondary ionization algorithm was used. In order to evaluate the computational efficiency of the new tree-based secondary ionization algorithm, the calculations as in Table 2 were run with the new SI solver enabled. The resulting computational times for a single attosecond simulation timestep are shown in Table 3. The code performance improves with increasing number of particles. It should also be emphasized that both secondary ionization algorithms, the default one and the tree-code based one, yield the same results, i.e., the resulting numbers are bit-wise identical. We have also observed that the effect of the new secondary ionization solver on the overall duration of a single simulation timestep is stronger in larger samples, when a tree-based Coulomb solver is also used.

This can be explained through a synergy between the two XMDYN algorithmic extensions. Generally, if one applies in a code a sequence of two algorithms, and if each of them has a complexity of $∼O(N^{2})$, then the overall complexity is still $∼O(N^{2})$—where *N* denotes the number of simulated particles. When the complexity of the first algorithm is improved to ∼O(N·log(N)), then the overall code complexity still remains $∼O(N^{2})$ because of the unchanged complexity of the second algorithm. However, the computation time might become shorter because of the improvement of the first algorithm. Only, when the scaling of both algorithms is changed to O(N·log(N)), the overall code complexity changes to O(N·log(N)). This explains the behaviour we observe and report on in the paper.

## 4. Conclusions

In summary, with the tree-code implementation performed by us, the computational efficiency of the classical molecular dynamics tool, XMDYN, developed to describe interactions of large atomic assemblies with ultrafast X-ray pulses, significantly increased. The XMDYN code is also used as a module of the simulation platform SIMEX, enabling virtual experiments on single-particle imaging at the European XFEL. With the newly incorporated tree-algorithm Coulomb solver and the tree-based secondary ionization solver, the XMDYN code is now able to efficiently follow X-ray induced evolution of larger samples (containing at least a few hundred thousand atoms) in single-particle imaging studies.

In particular, the computational tests performed revealed that the tree-based Coulomb solver speeds up the code performance only for sufficiently large samples (>10,000 particles). The original ‘brute-force’ (non-tree) Coulomb solver remains more efficient for smaller samples. The performance of the PEPC Coulomb solver, newly implemented in XMDYN, depends not only on the sample size but also on the selected θ parameter and the number of tree-walker threads used.

In contrast, the newly developed secondary ionization tree algorithm always performs better than the original non-tree algorithm. Its efficiency increases with increasing number of particles, when compared with the non-tree algorithm.

This improvement of XMDYN opens new prospects for computational single-particle imaging experiments using the EuXFEL’s simulation platform SIMEX, enabling for the first time virtual experiments with ‘realistic-size’ samples giving a sufficiently high scattering signal, e.g., large protein systems or viruses that are of strong interest for XFEL structure determination. The improved SIMEX simulations are expected to guide future single-particle-imaging experiments at the EuXFEL for samples of realistic size.

## Figures and Tables

**Table 1:** Simulated water samples, with specified total number of atoms and ions (including all charge states), ions (of charge +1 and higher), and free electrons.

Sample	Atoms & Ions	Ions	Electrons (Free)
47 Å	9722	2102	2806
60 Å	20,534	4422	5881
75 Å	40,358	8717	11,603
100 Å	97,655	21,087	28,084
150 Å	328,475	67,828	87,369

**Table 2:** Computational cost of a single attosecond timestep in XMDYN for samples of different size (first column) when using Coulomb brute-force scheme (second column), single threaded Coulomb PEPC tree code (third column), and the 4 threaded Coulomb PEPC solver (fourth column). The secondary ionization solver used in the calculation was the default one, i.e., the non-tree solver.

Sample	Brute [s]	Tree [s]	Tree (4 Threads) [s]
47 Å	0.444	0.956	0.300
60 Å	2.00	2.82	0.940
75 Å	7.82	6.26	2.44
100 Å	44.9	23.2	11.5
150 Å	464.1	123.4	94.1

**Table 3:** Computational cost of a single attosecond timestep in XMDYN, when using our new tree-based algorithm for calculation of secondary ionization. It was calculated for samples of different size (first column) when using Coulomb brute-force scheme (second column), single threaded Coulomb PEPC tree code (third column), and the 4 threaded Coulomb PEPC solver (fourth column).

Sample	Brute [s]	Tree [s]	Tree (4 Threads) [s]
47 Å	0.411	0.911	0.289
60 Å	1.80	2.56	0.760
75 Å	6.96	5.36	1.64
100 Å	39.5	17.7	6.00
150 Å	410.0	62.5	34.0

**Figure A1:**
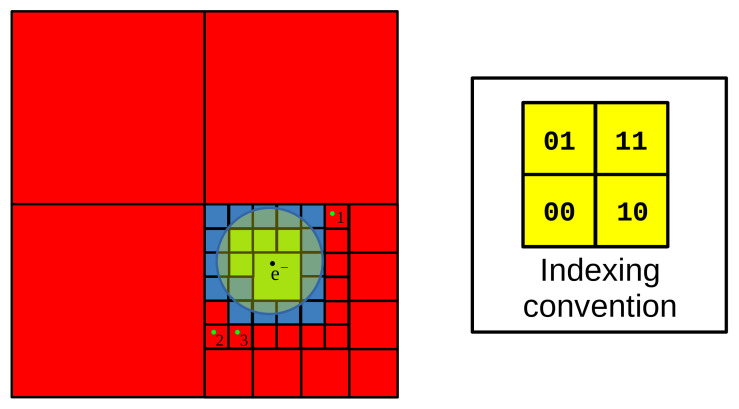
Illustration for recursive division of 2D volume into a Barnes-Hut tree. Each square can be subdivided into four smaller squares. These subdivisions form nodes of the tree. Each subdivision can be identified by two bits xy (as seen on the right plot), with values indicating in which of the four sub-squares the atom is located. For example, if the atom 1 is in the lower right square, it gets 10, as the most significant pair. After the next division, it obtains the index 11 etc. In the four-level tree, the atom 1 gets the 8-digit binary identification number 10|11|01|11. Similarly, the atom 2 gets 10|00|01|00, and the atom 3 gets 10|00|01|10. Atoms and ions are potential candidates for getting ionized by the electron e, if they are located within a predefined distance *d*, visualized by the circle.

## Data Availability

Data are available from the corresponding authors upon reasonable request.

## Abbreviations

The following abbreviations are used in this manuscript:

XFEL	X-ray free-electron laser
SPI	Single-particle imaging
LCLS	Linac Coherent Light Source
SIMEX	Simulation of Experiments at Advanced Laser Light Sources
XMDYN	Molecular-dynamics- and Monte-Carlo-based code for modelling X-ray driven dynamics in complex systems
XATOM	Atomic structure calculation tool
RE	Recombination
SI	Secondary ionization
MC	Monte Carlo
MD	Molecular dynamics
PEPC	Pretty Efficient Parallel Coulomb-solver
FWHM	Full width at half maximum

## Appendix A

At each time step, a Barnes-Hut tree is created for atoms and ions. An orthorhombic simulation box is defined so as to contain all the particles. Principally, the simulation box is then subdivided into eight orthorhombic sub-boxes (geometrically similar to their parent box). This occurs recursively, multiple times up to a maximum depth level, creating an oct-tree. Each atom or ion gets an identification number indicating (at each level) its position in the tree in the following way: each sub-box is identified by three bits xyz (each taking a value of 0 or 1) indicating in which part of the box (0= lower, 1= upper) the corresponding particle is located. These bit triplets are concatenated one after another, starting from the original box size, until the smallest division occurs. This chain forms the identification number for a particle position in a tree in a binary representation. The maximum depth level is based on the total number of atoms and ions. For illustrative purposes, a division of 2D volume into a tree is shown in Figure A1. In this case only two bits xy are required to represent a division. For example, the atom 1 lies in the lower right quadrant after the first division. Therefore, it gets the index 10 as the most significant pair. After the next division, it is located in the upper right quadrant, so the next pair is 11 etc. After the final division, joining the pairs, the binary identification number is 10110111. The atomic array is then sorted, using the standard quicksort algorithm, according to the identification number. A supporting structure representing the tree is built, in order to allow for an efficient searching. This algorithm is based on Ref. [29] with some modifications mentioned in Section 2.4 and with different supporting structures which facilitate searching. The overall computational complexity of the tree building is $O(N_{a}\cdot log\left(N_{a}\right))$, where $N_{a}$ is the number of atoms and ions. However, the benchmarking of the tree-building step showed that it has a negligible time cost, when compared to the subsequent tree searching or to other computations done outside the secondary ionization module. The most computationally expensive procedure of the secondary ionization module is actually the search of the closest atom or ion, performed for each electron. For each electron, it requires $O(log\left(N_{a}\right))$ computations. Therefore, for all electrons, it is, $O(N_{e}\cdot log\left(N_{a}\right))$, where $N_{e}$ is the electron number. With the tree building added, one gets the overall computational complexity of the algorithm equal to $O(\left(c_{1}\cdot N_{a}+c_{2}\cdot N_{e}\right)\cdot log\left(N_{a}\right))$ with some numerical coefficients $c_{1}$ and $c_{2}$. However, as it was mentioned before, the coefficient $c_{1}$ turns out to be very small. Atoms and ions are considered as potential candidates for getting ionized by an electron, if they stay within a predefined distance *d* from the electron, with *d* being derived from the secondary ionization cross section. This condition defines a sphere with the electron at its center. The recursive search for a candidate atom or ion in the tree contains the following steps at each level (see Figure A1):

(i) skip all boxes without intersection with the sphere (illustrated by red squares),
(ii) select all atoms and ions from boxes contained fully by the sphere (green squares) as candidates,
(iii) if a box has an intersection with the sphere but the sphere does not contain it entirely, then the box is subdivided and searched recursively. If this occurs after the final division (blue squares), the atoms and ions are searched one after another, in order to check if they lie within the sphere.
